# Responsiveness to bepridil predicts atrial substrate in patients with persistent atrial fibrillation

**DOI:** 10.1002/joa3.12492

**Published:** 2021-01-04

**Authors:** Daisuke Yakabe, Yusuke Fukuyama, Masahiro Araki, Toshihiro Nakamura

**Affiliations:** ^1^ Department of Cardiology Clinical Research Institute National Hospital Organization Kyushu Medical Center Fukuoka City Japan

**Keywords:** atrial fibrillation, atrial remodeling, bepridil hydrochloride, catheter ablation, low voltage zone

## Abstract

**Background:**

The low voltage zone (LVZ) detected with three‐dimensional electroanatomical mapping is a surrogate marker of atrial scar in patients with persistent atrial fibrillation (PeAF) and is associated with poor clinical outcomes after catheter ablation. However, fewer studies have reported the relationship between responsiveness to antiarrhythmic drugs and the LVZ.

**Methods:**

We retrospectively analyzed 76 patients who underwent catheter ablation for PeAF at our center. Rhythm control with bepridil was initiated before ablation in all patients, and electrical cardioversion was performed in cases of failure to restore sinus rhythm with bepridil alone. Patients with successful sinus restoration with bepridil alone (≤200 mg/d) were defined as “responders”, while those who required electrical cardioversion as well were defined as “non‐responders”. We compared the LVZ ratio (ratio of the LVZ surface area to the left atrium surface area on three‐dimensional electroanatomical mapping) and the recurrence‐free rate after ablation between the two groups.

**Results:**

Of the 76 patients, 48 (63.2%) were responders to bepridil. The median LVZ ratio was significantly lower in the responder group than in the nonresponder group (7.5% vs 14.0%, *P* = .009). Multivariate analysis revealed that response to bepridil was an independent predictor of normal voltage (*P* = .02, odds ratio = 0.20, 95% confidence interval = 0.04‐0.76). The recurrence‐free rate at 1 year after catheter ablation was significantly higher in the responder group than in the nonresponder group (87.1% vs 62.3%, *P* = .03).

**Conclusions:**

Response to bepridil is a marker of normal voltage in electroanatomical mapping and is significantly associated with better clinical outcomes after catheter ablation.

## Graphical abstract

Response to bepridil hydrochloride in patients with persistent atrial fibrillation is associated with normal voltage in the three‐dimensional electroanatomical mapping and better clinical outcome after catheter ablation.

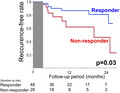

## 1 INTRODUCTION

Atrial fibrillation (AF) refers to progressive arrhythmia through electrical and structural remodeling.^1^, ^2^ Its pathophysiology can involve a change in various ionic currents. Particularly, intracellular calcium overload is one of the major mechanisms in the pathophysiology of AF.^3^ As AF progresses, structural remodeling such as collagen accumulation, fibroblast proliferation, and atrial fibrosis can occur. In clinical practice, the consequences of these remodeling processes are recognized as a late gadolinium enhancement on cardiac magnetic resonance imaging (MRI) and a low voltage zone (LVZ) on three‐dimensional electroanatomical mapping.^4^, ^5^, ^6^, ^7^, ^8^ These are strongly associated with a recurrence of AF after pulmonary vein isolation(PVI)‐based catheter ablation.^8^

Bepridil hydrochloride is a class IV oral antiarrhythmic drug (AAD) that is mainly effective in blocking calcium and potassium currents. Bepridil is recommended as the standard AAD for rhythm control in persistent AF (PeAF) in the Japanese pharmacotherapy guidelines for AF.^9^ Bepridil can reverse electrical remodeling in the canine model,^10^ and some studies have revealed that pretreatment with bepridil and catheter ablation is effective for PeAF in clinical practice.^11^, ^12^

We have previously reported the efficacy of a hybrid therapy involving pretreatment with bepridil and PVI with cryoballoon for PeAF.^12^ In that study, patients who were successfully restored to sinus rhythm (SR) with bepridil had better clinical outcomes after PVI. However, there is a knowledge gap between this "response to bepridil" and the clinical outcome after catheter ablation. We hypothesized that response to bepridil can help detect patients with early‐stage PeAF. Thus, this study aimed to examine whether responsiveness to bepridil could predict the presence of LVZ and the clinical outcomes of catheter ablation in patients with PeAF.

## 2 METHODS

### 2.1 Patient selection and protocol

We conducted a retrospective observational study involving 76 patients with PeAF who underwent an index catheter ablation at our center from January 2018 to February 2020. According to expert consensus, PeAF is defined as AF lasting for more than one week, and long‐standing PeAF is defined as AF lasting for more than one year.^13^ The exclusion criteria were as follows: patients with (1) previous history of catheter ablation and cardiac surgery, (2) severe valve disease, (3) end‐stage renal disease on hemodialysis, (4) intracardiac thrombi, and (5) a corrected QT interval (QTc) of 440 ms or greater, calculated with the Bazzett formula before bepridil administration. Based on the patients’ clinical parameters, the estimated glomerular filtration rate (eGFR) was calculated using the Cockcroft–Gault equation. The APPLE score, reported as the predictive factor of the presence of LVZ in electroanatomical mapping and recurrence after catheter ablation, was also calculated (1 point each for age >65 years, PeAF, eGFR < 60 mL/min/m^2^, left atrial diameter [LAD] ≥43 mm, and ejection fraction [EF] <50%).^14^, ^15^ Data from an echocardiographic examination performed within three months before catheter ablation were used for the analysis. In cases where echocardiography was performed more than once (eg, before rhythm control and just before catheter ablation), the data from the examination conducted just before catheter ablation were included for the analysis.

The protocol of rhythm control before catheter ablation was as follows: Bepridil administration (100 mg per day) was initiated at the outpatient clinic after confirming that the patients had agreed to undergo catheter ablation and had already been treated with an oral anticoagulant for at least 4 weeks. When AF persisted after initiating bepridil, the dose was increased by 50 mg per day every 2 to 4 weeks while the QTc was monitored by a 12‐lead electrocardiogram (ECG) and an automatic measurement system (Physiological Examination System EFS‐8800, Fukuda Denshi C. Ltd., Tokyo, Japan). The maximum dose of bepridil was 200 mg per day. If the QTc interval was prolonged, the dose of bepridil was reduced according to the physician's discretion. When SR could not be confirmed even after the maximum dose of bepridil administration, the patient was admitted and additional electrical cardioversion (ECV) was conducted after exclusion of intracardiac thrombi with transesophageal echocardiography. Catheter ablation was performed in all patients, regardless of whether SR was restored with this protocol.

The protocol for this study was approved by the Ethics Committee of our center, and it conformed to the provisions of the Declaration of Helsinki. Informed consent was obtained from all the patients.

### 2.2 Mapping protocol and analysis

Bepridil was discontinued 1‐2 weeks before ablation. Warfarin was continued if the prothrombin time‐international normalized ratio was appropriate, while direct oral anticoagulant was skipped on the day of ablation. Contrast‐enhanced computed tomography was performed to confirm the anatomy of the pulmonary veins (PVs) and the absence of an intracardiac thrombus. The patients were then sedated with dexmedetomidine. Unfractionated heparin was administered after femoral vein puncture, and an atrial septal puncture was performed. A high‐resolution voltage map of the LA was created using a multi‐electrode mapping catheter (PENTARAY catheter, Biosense Webster, Inc, Diamond Bar, CA, USA) and a three‐dimensional electroanatomical mapping system (CARTO3, Biosense Webster, Inc). When patients presented to the laboratory in SR, mapping was performed under atrial pacing by placing a distal electrode into the coronary sinus (CS) at a sinus rate of more than 10‐20 ppm. The electrograms were filtered at 16‐500 Hz from the mapping and reference catheters (5th–6th electrode of CS). In case of AF, the voltage map was created after ECV. If SR could not be maintained even after cardioversion, the voltage map was created after PVI plus ECV. There were no patients for whom a voltage map could not be created with this method. Continuous point acquisition was performed using the CONFIDENSE Module (Biosense Webster, Inc). The detailed settings were as follows: cycle length filtering, 5%; local activation time stability, 5 ms; position stability, 3 mm; and density, 1 mm. The window of interest was set to +5‐10 ms from the CS reference to avoid mapping pacing artifacts and +15 ms from the beginning of the QRS complex to avoid mapping signals from the ventricle. Low voltage potentials were also confirmed by a 3.5 mm‐tip irrigation contact‐sensing ablation catheter (ThermoCool Smarttouch SF, Biosense Webster, Inc). Tissue proximity indicator was also used to determine the mapping electrode's proximity to the cardiac tissue in some cases. The LVZ was defined as an area of mapping points with a bipolar voltage amplitude (peak to peak) < 0.5 mV.^5^, ^6^, ^7^, ^8^ The LA area was measured after the exclusion of the PVs and the left atrial appendage. Then, the LVZ surface area was measured by two blinded examiners, and the average value was adopted for analysis. The presence of LVZ was defined by a ratio of the LVZ area to the LA surface area (ie, the LVZ ratio) of greater than 20%. Furthermore, we also used the Utah fibrosis classification to diagnose the severity of the LVZ ratio, as follows: LVZ ratio <5% (Stage I), 5%–20% (Stage II), 20%–30% (Stage III), and >40% (Stage IV).^4^

**FIGURE 1:**
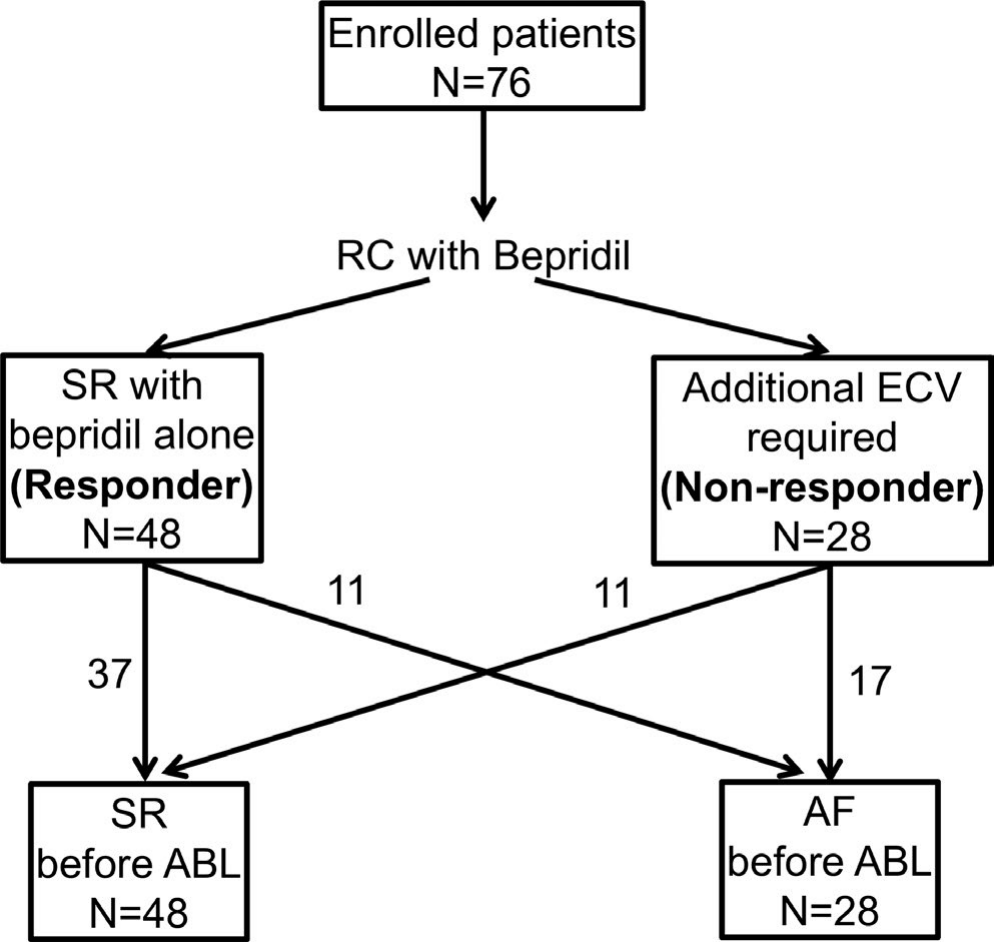
Patient flow until catheter ablation. ABL, ablation; AF, atrial fibrillation; ECV, electrical cardioversion; RC, rhythm control; SR, sinus rhythm

Based on the responsiveness to bepridil before catheter ablation, the patients were divided into two groups: the responder group (in whom SR was successfully restored with bepridil alone) and the nonresponder group (in whom SR was not restored with bepridil, and ECV was required). The patients who were successfully restored to SR after starting bepridil, but presented with AF at the next outpatient clinic or laboratory visit, were also classified into the responder group; even when SR recovery by bepridil alone was confirmed only once. In addition, the patients were also divided into two groups based on the result of our rhythm control protocol before ablation; the SR group when SR was successfully maintained until catheter ablation, and the AF group when AF was recorded on ECG even once until ablation after rhythm control protocol (Figure 1).

### 2.3 Ablation strategies and follow‐up

PVI was performed in all patients. Furthermore, cavotricuspid isthmus ablation was performed when common atrial flutter was documented before and during ablation. An induction test of non‐PV foci or atrial tachycardia using isoproterenol infusion and rapid atrial pacing was not conducted. Superior vena cava isolation and posterior wall isolation (PWI) were performed in some patients at the discretion of the operators. Substrate modifications, such as other linear ablations, LVZ‐guided ablation, and complex fractionated atrial electrogram ablation, were not performed. Administration of oral anticoagulant and bepridil was restarted at the same doses after catheter ablation. Bepridil was discontinued 3‐6 months after catheter ablation. The blanking period was defined as the period within 3 months after ablation, and recurrence of atrial tachyarrhythmia (ATA) during this period was excluded for the event.^13^ The 12‐lead ECG or the holter ECG recordings were performed at 3, 6, 12, and every 12 months. Additional holter and portable ECGs were used to detect recurrence of ATA when the patients experienced AF‐related symptoms. Recurrence of ATA was defined as an ATA lasting for more than 30 seconds. The patients in whom bepridil was continued for 6 months or more after catheter ablation were also regarded to have experienced a recurrence.

### 2.4 Statistical analysis

Statistical analysis was conducted using the JMP 14.2.0 software (SAS Institute Inc, Cary, NC, USA). Continuous variables were expressed as means ± standard deviation (SD) or median with interquartile ranges (IQR). Categorical variables were expressed as percentages. The clinical characteristics were compared using the Student's *t* test or the Mann–Whitney *U* test for continuous variables and the Fisher's exact test for categorical variables. A logistic regression model was used to identify the independent predictors of the presence of LVZ (the LVZ ratio ≥20%). In a multivariate analysis, we used two variables, namely “response to bepridil” and an additional variable that was reported as a predictor in previous studies.^14^, ^15^, ^16^, ^17^, ^18^ The recurrence‐free rate after catheter ablation was compared between the responder and nonresponder groups using the Kaplan‐Meier analysis and log‐rank test. Cox proportional hazard analysis was used to identify the predictors of recurrence with two models (model 1 includes the clinical background and model 2 includes the ablation procedures). In the multivariate analysis, the variables of age, eGFR, LAD, and EF were not used because the APPLE score included all of them for calculation.^14^, ^15^ The correlation analysis between the pacing cycle length (PCL) during voltage mapping and the LVZ ratio was also performed. All statistical analyses were two‐tailed, and *P* < .05 was considered to be statistically significant.

## 3 RESULTS

### 3.1 Patients’ characteristics

The patients' background characteristics are shown in Table 1. Among the 76 patients analyzed, including the 47 (62.7%) with long‐standing PeAF, the median duration of AF was 1.2 years (IQR: 0.7‐3.0 years). Bepridil was used at an average dose of 144.7 ± 35.2 mg per day, and SR was successfully restored in 2.4 ± 1.3 months in 48 patients (63.2%) after starting bepridil (the responder group). The remaining 28 patients included those who required additional ECV to achieve SR (the nonresponder group). Seven patients had a prolonged QTc interval (>500 ms), and six of these were administered bepridil at a dose of 200 mg per day. All seven patients required a reduction in the dose; however, no adverse events, such as torsades de pointes and thromboembolism, were noted. After rhythm control, SR was maintained in 48 patients until catheter ablation (Figure 1). AF recurred after rhythm control in 28 patients (11 in the responder group and 17 in the nonresponder group); however, in seven of these, the AF was paroxysmal and did not persist.

**TABLE 1:** Patients’ background characteristics (according to all patients as well as each group)

	All patients	Responder group	Nonresponder group	*P* value
N	76	48	28	
Patients' background characteristics				
Age (y)	68.1 ± 9.7	68.0 ± 9.8	68.3 ± 9.6	.9
Female sex, n (%)	19 (25.0)	13 (27.1)	6 (21.4)	.8
BMI (kg/m^2^)	23.8 ± 4.1	23.4 ± 3.9	24.4 ± 4.4	.3
AF duration ≥ 1 year, n (%)	47 (62.7)	25 (53.2)	22 (78.6)	**.047**
CHADS_2_ score	1.9 ± 1.5	1.9 ± 1.5	1.9 ± 1.7	1.0
eGFR (mL/min/m^2^)	70.9 ± 30.6	68.3 ± 31.4	75.4 ± 29.2	.3
EF (%)	60.9 ± 11.7	59.7 ± 12.8	63.1 ± 9.3	.2
LAD (mm)	42.8 ± 6.5	42.4 ± 7.0	43.4 ± 5.6	.5
APPLE score	2.9 ± 1.0	2.9 ± 1.0	2.9 ± 1.0	.9
Dose of bepridil (mg per day)	144.7 ± 35.2	134.4 ± 36.0	162.5 ± 25.9	**.0005**
Maximum QTc interval (ms)	468.2 ± 24.8	466.6 ± 24.2	471.0 ± 26.1	.5
Voltage mapping				
LA surface area (cm^2^)	142.9 ± 29.7	141.8 ± 29.5	144.6 ± 30.5	.7
LVZ surface area (cm^2^)	13.5 [7.4‐25.3]	11.0 [6.4‐20.4]	19.8 [12.3‐31.7]	**.005**
LVZ ratio (%)	10.5 [5.2‐16.8]	7.5 [4.7‐13.6]	14.0 [9.9‐20.9]	**.009**
LVZ ratio ≥20%, n (%)	12 (15.8)	4 (8.3)	8 (28.6)	**.03**
LVZ stage I	18 (23.7)	14 (29.2)	4 (14.3)	
LVZ stage II	46 (60.5)	30 (62.5)	16 (57.1)	
LVZ stage III	9 (11.8)	4 (8.3)	5 (17.9)	
LVZ stage IV	3 (4.0)	0 (0.0)	3 (10.7)	
Ablation procedures				
PVI, n (%)	76 (100.0)	48 (100.0)	28 (100.0)	1.0
SVCI, n (%)	19 (25.0)	12 (25.0)	7 (25.0)	1.0
CTI ablation, n (%)	8 (10.5)	4 (8.3)	4 (14.3)	.5
PWI, n (%)	11 (14.5)	7 (14.6)	4 (14.3)	1.0

Note

Data are represented as mean ± standard deviation, median [interquartile range], or n (%). Values in bold are significant.

Abbreviations: AF, atrial fibrillation; BMI, body mass index; CTI, cavotricuspid isthmus; EF, ejection fraction; eGFR, estimated glomerular filtration rate; LA, left atrium; LAD, left atrium diameter; LVZ, low voltage zone; PVI, pulmonary vein isolation; PWI, posterior wall isolation; SVCI, superior vena cava isolation.

A total of 1780 ± 689 points in a voltage map were acquired during electroanatomical mapping. The mean PCL was 753.2 ± 119.3 ms The mean LA surface area was 142.9 ± 29.7 cm^2^, the median LVZ surface area was 13.5 cm^2^ [IQR: 7.4‐25.3 cm^2^], and the median LVZ ratio was 10.5% [IQR: 5.2%–16.8%].

### 3.2 Comparison between responder and nonresponder groups

**FIGURE 2:**
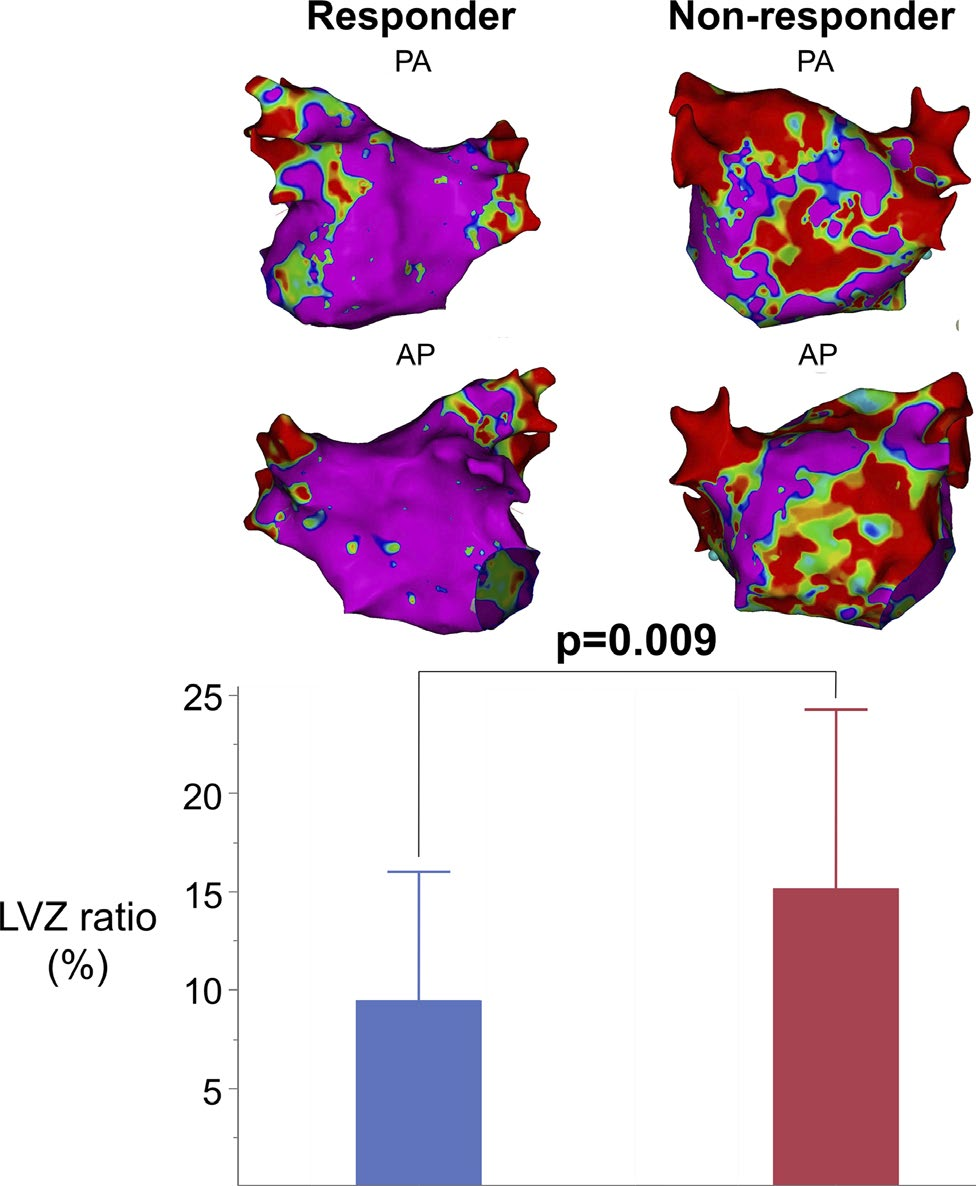
Voltage map of the responder and nonresponder groups. This figure includes the voltage maps of the two representative cases (above). The map on the left is of a 60‐year‐old male who was successfully restored to sinus rhythm with 100 mg per day of bepridil. The LVZ ratio was 2.6%. The map on the right is of a 72‐year‐old female who consumed 200 mg per day of bepridil and required electrical cardioversion for achieving sinus rhythm. The LVZ ratio was 38.2%. AP, anterior‐posterior view; LVZ, low voltage zone; PA, posterior anterior view

A comparison between the two groups is shown in Table 1 and Figure 2. There were no differences in the patients’ background characteristics between both groups, except for the number of patients with long‐standing PeAF (responder vs nonresponder groups: 53.2% vs 78.6%; *P* = .047) and the daily dose of bepridil (responder vs nonresponder groups: 134.4 ± 36.0 mg vs 162.5 ± 25.9 mg; *P* = .0005). In the electroanatomical mapping, the median LVZ ratio was significantly lower in the responder group than in the nonresponder group (7.5% vs 14.0%, *P* = .009). More than 90% of the patients in the responder group presented with Utah Stages I and II (LVZ ratio ≤20%), and there were no patients with Stage IV.

### 3.3 Predictors of LVZ

Univariate analysis (Table 2) revealed that an eGFR < 60 mL/min/m^2^ (*P* = .048, OR = 3.57, 95% confidence interval [CI] = 1.01‐14.57) and a higher APPLE score (*P* = .02, OR = 2.22 per 1, 95% CI = 1.15‐4.74) were the predictive factors of an LVZ ratio of ≥20%. Response to bepridil predicted a lower LVZ ratio (*P* = .02, OR = 0.23, 95% CI = 0.06‐0.81). Multivariate analysis also revealed that response to bepridil was associated with a lower LVZ ratio.

**TABLE 2:** Predictors of the presence of LVZ

	*P* value	OR (95% CI)
Univariate		
Age†	.2	1.05 (0.98‐1.15)
Female sex	.5	0.55 (0.08‐2.34)
BMI†	.3	0.91 (0.75‐1.07)
AF duration ≥ 1 year	.08	2.00 (0.83‐9.54)
CHADS_2_ score†	.1	1.37 (0.93‐2.03)
eGFR < 60 mL/min/m^2^	**.048**	**3.57 (1.01‐14.57)**
EF†	.3	0.97 (0.92‐1.02)
LAD†	.7	1.01 (0.92‐1.11)
APPLE score†	**.02**	**2.22 (1.15‐4.74)**
Responder	**.02**	**0.23 (0.06‐0.81)**
Multivariate (model 1)		
Responder	**.02**	**0.23 (0.06‐0.83)**
Female sex	.5	0.60 (0.08‐2.73)
Multivariate (model 2)		
Responder	**.04**	**0.25 (0.06‐0.93)**
AF duration ≥ 1 year	.7	1.38 (0.33‐7.04)
Multivariate (model 3)		
Responder	**.02**	**0.20 (0.04‐0.76)**
APPLE score†	**.01**	**2.40 (1.19‐5.58)**

Note

Values in bold are significant. † indicates per 1 unit odds ratio.

Abbreviations: AF, atrial fibrillation; BMI, body mass index; CI, confidence interval; EF, ejection fraction; eGFR, estimated glomerular filtration rate; LAD, left atrium diameter; OR, odds ratio.

### 3.4 Catheter ablation and clinical outcomes

Successful PVI was achieved in all patients (Table 1). There were no differences in the adjunctive procedures between the two groups. The median LVZ ratio in the posterior wall of the LA was similar between patients with PWI and non‐PWI (2.1% vs 2.0%, *P* = .6). One patient experienced inguinal hematoma at the puncture site after catheter ablation; however, it was treated without the need for any invasive treatment.

**FIGURE 3:**
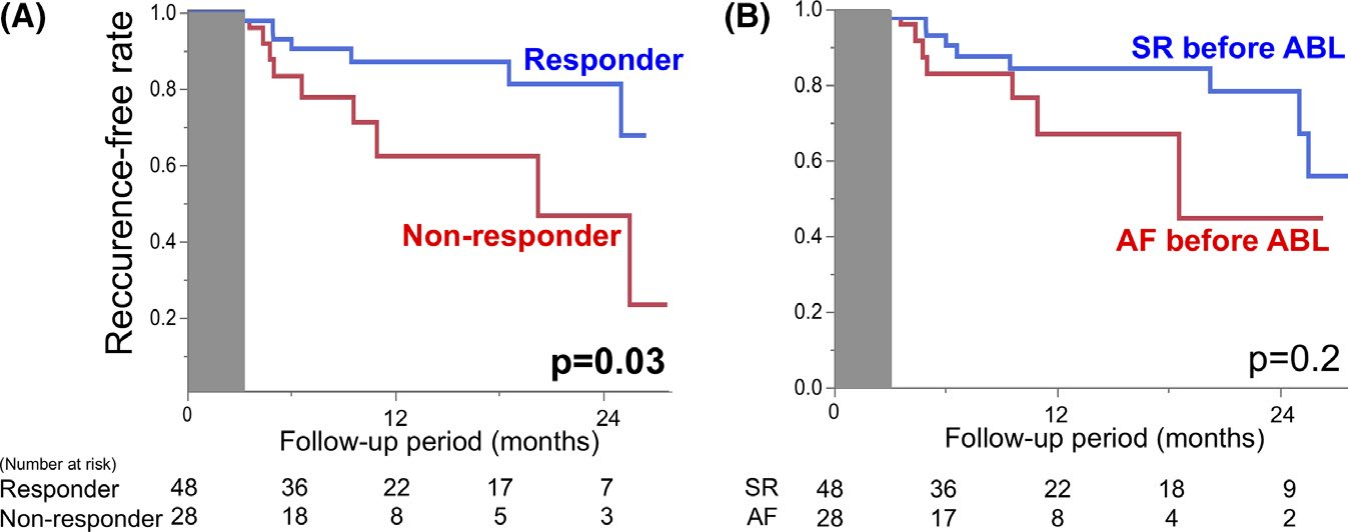
Recurrence‐free rates after catheter ablation. The gray zone indicates the blanking period. (A) A comparison of the responsiveness to bepridil revealed that the recurrence‐free rate after catheter ablation was significantly lower in the nonresponder group than in the responder group (*P* = .03). (B) On the other hand, there were no significant differences between the sinus rhythms before and after ablation (*P* = .2). ABL, catheter ablation; AF, atrial fibrillation; SR, sinus rhythm

Bepridil was discontinued in 73 patients within 6 months after catheter ablation. In the remaining three patients (one in the responder group and two in the nonresponder group), bepridil was continued even after 6 months, and these cases were classified as having AF recurrence. The recurrence‐free rate was plotted using Kaplan–Meier curves (Figure 3A). The recurrence‐free rate at 1 year after catheter ablation was significantly higher in the responder group than in the nonresponder group (87.1% vs 62.3%, *P* = .03). Cox proportional hazard analysis revealed that response to bepridil was associated with a better clinical outcome after catheter ablation (Table 3). Regarding the rhythm before catheter ablation, there were no significant differences between the SR group and the AF group (*P* = .2, Figure 3B).

**TABLE 3:** Predictors of recurrence after catheter ablation

	Univariate		Multivariate (model 1)		Multivariate (model 2)	
	*P* value	HR (95% CI)	*P* value	HR (95% CI)	*P* value	HR (95%CI)
Age†	.5	1.02 (0.97‐1.08)	‐	‐	‐	‐
Female sex	.7	0.81 (0.23‐2.86)	‐	‐	‐	‐
BMI†	1.0	1.00 (0.88‐1.11)	‐	‐	‐	‐
AF duration ≥ 1 year	.9	1.10 (0.34‐3.55)	.6	1.37 (0.37‐5.11)	‐	‐
CHADS_2_ score†	.4	1.13 (0.84‐1.51)	‐	‐	‐	‐
eGFR < 60 mL/min/m^2^	.7	1.25 (0.43‐3.60)	‐	‐	‐	‐
EF†	.7	0.99 (0.96‐1.03)	‐	‐	‐	‐
LAD†	**.03**	**1.08 (1.01‐1.14)**	**‐**	**‐**	**‐**	**‐**
APPLE score†	**.02**	**1.87 (1.12‐3.26)**	**.04**	**1.79 (1.03‐3.26)**	**‐**	**‐**
Responder	**.04**	**0.35 (0.13‐0.96)**	**.04**	**0.34 (0.11‐0.98)**	**.01**	**0.26 (0.09‐0.75)**
LVZ ratio ≥ 20%	**.01**	**4.22 (1.43‐12.21)**	.2	2.30 (0.71‐7.47)		
SVCI	.3	0.48 (0.11‐2.16)	‐	‐	.3	0.45 (0.10‐2.07)
CTI ablation	.1	4.08 (0.89‐12.06)	‐	‐	.06	4.16 (0.91‐12.01)
PWI	.2	0.26 (0.03‐1.97)	‐	‐	.1	0.2 (0.03‐1.67)

Note

Values in bold are significant. † indicates per 1 unit hazard ratio.

Abbreviations: AF, atrial fibrillation; BMI, body mass index; CI, confidence interval; CTI, cavotricuspid isthmus; EF, ejection fraction; eGFR, estimated glomerular filtration rate; HR, hazard ratio; LAD, left atrium diameter; LVZ, low voltage zone; PWI, posterior wall isolation; SVCI, superior vena cava isolation.

## 4 DISCUSSION

### 4.1 Main findings

The summary of this study's findings is as follows: (1) In 63.2% of the patients SR was successfully restored with bepridil alone, (2) The LVZ ratio was significantly lower in the responder group than in the nonresponder group, and (3) Response to bepridil was an independent predictor of a lower LVZ ratio and better clinical outcomes after catheter ablation.

### 4.2 Pathophysiology of AF and therapeutic effect of bepridil

Electrical remodeling, as part of AF pathophysiology, includes changes in various ionic currents, connexin lateralization, and intracellular calcium handling. Intracellular calcium overload plays a major role in AF initiation and maintenance.^2^, ^3^ Tachycardia causes a calcium overload in the myocardium, thereby resulting in early or delayed after depolarization.^3^ In addition, a shortened action potential duration reduces the wavelength and causes numerous sustained re‐entry circuits. Decrease in the inward sodium current and connexin lateralization results in a local conduction delay and is associated with AF maintenance.^3^ Furthermore, the calcium overload can activate and proliferate fibroblasts and increase the extracellular matrix, causing structural remodeling.^19^

Conversely, bepridil exerts various antiarrhythmic effects, including calcium blockade to suppress the intracellular calcium overload and potassium blockade to prolong the action potential duration and the atrial refractory period.^20^, ^21^, ^22^ The L‐type calcium channels are down‐regulated in the fibrillated atrial myocardium; however, bepridil can normalize the L‐type calcium current and can induce an electrical reverse remodeling.^10^ In clinical practice, bepridil is highly effective in sinus restoration for PeAF. Nakazato et al reported that 58% of the patients with PeAF were successfully restored to SR in a mean of 2.4 months.^23^ In a comparative study against amiodarone, bepridil had a higher success rate in restoring SR (bepridil vs amiodarone = 85% vs 35%).^24^ This success rate was similar to that observed in this study. However, bepridil can block the potassium current; therefore, careful monitoring with 12‐lead ECG is required to avoid excessive QT prolongation and torsade de pointes. In our study, seven patients (9.2%) had a longer QTc interval (> 500 ms, maximum QTc interval was 523 ms) and required a reduction in the bepridil dose; however, no patients presented with excessive QT prolongation or torsade de pointes.

### 4.3 Response to bepridil and atrial substrate

The presence of LVZ is indicative of advanced AF and is reported to be a predictive factor of recurrence after catheter ablation.^8^ However, not all patients with PeAF have LVZ. LVZ was found in 27% to 54% of the patients with PeAF,^14^, ^15^, ^16^ and several factors (such as an increased age, female sex, LA volume, and coexistence of sinus node dysfunction) and clinical scores (such as the APPLE score) were reported as predictors of LVZ.^14^, ^16^, ^17^, ^18^ Our results also demonstrated the usefulness of the APPLE score. However, response to bepridil was independently associated with the normal voltage of the LA. To our knowledge, this is the first study evaluating the relationship between the responsiveness to AADs and the extent of atrial substrate. Response to bepridil may be a sensitive marker for detecting early‐stage PeAF.

There are two possible reasons to explain why responders to bepridil had a lower LVZ. First, bepridil may be ineffective in patients with advanced PeAF. Prolongation of the atrial fibrillation cycle length (AFCL) on surface ECG after administration of bepridil is reported to be a predictor of SR restoration.^25^ The AFCL also reflects the atrial refractory periods and temporal excitable gaps, and is inversely proportional to the number of sources sustaining AF.^26^ Therefore, there may be a greater extent of pathological arrhythmogenic substrates in the nonresponder group, which can be reflected as the presence of LVZ. Second, AADs including bepridil may induce reverse remodeling. Several clinical studies support the latter hypothesis. Khan et al reported similar results using dofetilide; they also revealed that the P wave duration was shortened after successful sinus restoration with dofetilide.^27^ They speculated that dofetilide could induce electrical reverse remodeling before catheter ablation. We also reported similar results using bepridil before cryoballoon ablation.^12^ In addition, Rivard et al reported that rigorous rhythm control (AAD with ECV) before catheter ablation could shorten the procedure time of the stepwise ablation procedure.^28^ Not only AADs, but also ECV can induce reverse remodeling.^29^ Therefore, we speculate that rhythm control may have induced electrical reverse remodeling and reduced the LVZ ratio in some cases of the responder group. However, it is difficult to verify this speculation, because this was not a longitudinal study (eg, a study to evaluate the voltage map before and after administration of bepridil in the same patients). In addition, the maintenance of SR itself with rhythm control might affect our results. When comparing the clinical outcomes after ablation with respect to the maintenance of SR after rhythm control, the recurrence‐free rate tended to be higher in the group in which SR could be maintained than in the group in which AF recurred after rhythm control (Figure 3B). It has also been reported that an improvement of the LA function was achieved from rhythm control undertaken in advance, and was associated with a lower recurrence rate after catheter ablation.^30^ Thus, not only the responsiveness to bepridil, but also the maintenance of SR itself might have led to our results. To verify the bepridil‐specific effect, an additional comparative study with a control group that does not use bepridil (ie, receives ECV alone) is necessary.

### 4.4 Study limitations

There were several limitations in this study. First, this was a retrospective observational study in a single center with a relatively small population that was administered bepridil, a drug which is only available in Japan and France; therefore, other AADs such as amiodarone or dofetilide should be investigated. Second, voltage mapping was performed not during SR, but during atrial pacing at distal CS electrodes. The amplitude of the bipolar voltage was reported to be lower during atrial pacing from distal CS at a faster rate than that during SR^31^, ^32^; therefore, the degree of LVZ obtained by our mapping method may be enhanced. In our study, there was no correlation between PCL during voltage mapping and the LVZ ratio (*P* = .4, r = 0.09). This may be because the PCL in our study was relatively longer (PCL = 753.2 ± 119.3 ms) than that in a previous study (PCL = 300 ms).^32^ In addition, we also did not create the substrate map during AF, which may have been useful for evaluating the specific electrophysiological properties of AF.^33^ Third, asymptomatic recurrence might not be partially detected with holter and portable ECGs alone. Finally, it is difficult to discuss the ablation strategies in detail because of the retrospective study design. In order to solve these limitations, a multicenter, prospective comparative study with a unified method of mapping and ablation should be conducted.

## 5 CONCLUSIONS

Response to bepridil may be a sensitive marker of normal voltage during electroanatomical mapping in patients with PeAF. This finding can also predict better clinical outcomes after PVI‐based ablation.

## CONFLICT OF INTEREST

The authors declare no conflicts of interests for this article. The protocol for this study was approved by the Ethics Committee of our center on 22 April 2020 (Approval number: 20C009).
